# Analysis of the enablers of capacities to produce primary health care-based reforms in Latin America: a multiple case study

**DOI:** 10.1093/fampra/cmw038

**Published:** 2016-05-21

**Authors:** Ernesto Pablo Báscolo, Natalia Yavich, Jean-Louis Denis

**Affiliations:** 1 National Scientific and Technical Research Council (Consejo Nacional de Investigaciones Científicas y Técnicas, CONICET), Center for Interdisciplinary Studies (Centro de Estudios Interdisciplinarios), National University of Rosario (Universidad Nacional de Rosario), Rosario, Argentina; 2 Canada Research Chair on Governance and Transformation of Health Organizations and Systems, École Nationale d’Administration Publique, Montréal, Québec, Canada

**Keywords:** Health care quality, access, and evaluation, health care reform, leadership, organizational innovation, politics, primary health care

## Abstract

**Background:**

Primary health care (PHC)-based reforms have had different results in Latin America. Little attention has been paid to the enablers of collective action capacities required to produce a comprehensive PHC approach.

**Objective:**

To analyse the enablers of collective action capacities to transform health systems towards a comprehensive PHC approach in Latin American PHC-based reforms.

**Methods:**

We conducted a longitudinal, retrospective case study of three municipal PHC-based reforms in Bolivia and Argentina. We used multiple data sources and methodologies: document review; interviews with policymakers, managers and practitioners; and household and services surveys. We used temporal bracketing to analyse how the dynamic of interaction between the institutional reform process and the collective action characteristics enabled or hindered the enablers of collective action capacities required to produce the envisioned changes.

**Results:**

The institutional structuring dynamics and collective action capacities were different in each case. In Cochabamba, there was an ‘interrupted’ structuring process that achieved the establishment of a primary level with a selective PHC approach. In Vicente López, there was a ‘path-dependency’ structuring process that permitted the consolidation of a ‘primary care’ approach, but with limited influence in hospitals. In Rosario, there was a ‘dialectic’ structuring process that favoured the development of the capacities needed to consolidate a comprehensive PHC approach that permeates the entire system.

**Conclusion:**

The institutional change processes achieved the development of a primary health care level with different degrees of consolidation and system-wide influence given how the characteristics of each collective action enabled or hindered the ‘structuring’ processes.

## Introduction

Primary health care (PHC)-based reforms have been used to promote changes in health systems and services. These initiatives have fostered a transformation from health care models that are hospital-centred, curative, medical and specialized to models that focus on more comprehensive, family- and community-oriented care, with PHC as a strategy to coordinate health care across levels and providers (1).

In Latin America, these change processes emerged in the 1980s with the rise of democratic governments that empowered the participation of new social actors committed to the improvement of social determinants of health and health equity. The advances engendered by these processes to change from one health care model to another were uneven (2–6). While some countries moved towards a comprehensive PHC approach accompanied by radical institutional and organizational changes that resulted in significant health improvements, other countries were able to develop only the primary level of care or introduce programmes to improve maternal and child health and control infectious diseases. Although these latter processes favoured significant health improvements in specific populations and/or diseases, they were unable to produce the institutional and organizational changes required to reform and improve the health system as a whole (1–3).

The institutional process plays a fundamental role as a driver of organizational change through the introduction of regulatory mechanisms, structures, conceptions and values (7,8). The question that continues to be the subject of an unresolved policy and academic debate is how to produce institutional change (8,9). According to Hodgson (10), the triggers of institutional change depend on the capacities for collective action, in other words, the group of stakeholders who are advocating for institutional change.

Two approaches have analysed the enablers of collective action capacities to produce institutional change processes. One approach emphasizes the configuration of the collective action as the main enabler of capacities to manage institutional change processes (10). The other approach assumes that collective action capacities are modelled by new structures created during the trajectory of the institutional restructuring process (8). We hypothesize that collective action capacities consist of the dynamics between actors and structures, known as structuring, which underlie institutional change processes (11).

Our research question is how did structuring processes influence the achievement of different levels of advances towards developing a comprehensive PHC approach in three reform processes? More specifically, our objective is to analyse how structuring processes during three Latin American reforms aimed at the implementation of a comprehensive PHC approach enabled or hindered the collective action capacities needed to implement the changes envisioned.

## Methods

### Study design

We conducted a multicentre, longitudinal, retrospective multiple case study (12) between 2007 and 2010. The research was coordinated by an Argentinean team in charge of the overall coordination and analysis, the design of the data collection procedures and tools and the data collection in the Argentinean settings. A Bolivian team based in Cochabamba collected and analysed the local data.

### Units of analysis

Our units of analysis were the reforms implemented between 1990 and 2008 in the municipalities of Cochabamba (Department of Cochabamba, Bolivia); Vicente López (Province of Buenos Aires, Argentina); and Rosario (Province of Santa Fe, Argentina). We selected reforms that took place at the municipal level since the decentralization processes during the 1980s and 1990s in Latin America positioned the municipal level at the core of the implementation of these reforms (5,6). Table 1 provides basic information about each municipality’s socio-economic and health services characteristics.

**Table 1.** Sociodemographic and health services indicators for the municipalities under study

Municipality	Cochabamba	Vicente López	Rosario
Number of residents^a^	517024	274082	909397
Percentage of structurally poor population according to the unmet basic needs index^a^	33.8%	4.8%	13.5%
Percentage of population without health coverage^a^	75.1%	27.2%	39.1%
Ratio of public primary level of care centres per 10000 population without coverage^a,b^	0.7	2.6	2.3
Ratio of primary level of care providers per 1000 population without coverage^a,b^	0.3	1.2	1.4
Number of public hospitals^b^	5	6	14

Source: ^a^Argentina: National Population, Households, and Dwellings Census (13), 2001; Bolivia: National Population and Housing Census, 2001 (14).

^b^The list of health centres and hospitals provided by the Health Secretaries.

### Sampling rationale

To better understand the differences in the collective action capacities that led to different degrees of institutional change, we used a purposeful sampling strategy, to selected Latin American cases showing a maximum variation with regard to the PHC approaches achieved by the reforms (2–4). While in Europe and other wealthy developed countries PHC has primarily been viewed as the first level of health services for the entire population, in Latin America coexist a number of PHC conceptions and models. These models fall broadly into three main organizational approaches: (i) Selective PHC: focused in a limited number of high-impact services to target the most prevalent causes of child mortality and some infectious diseases. Although originally considered an interim strategy, this approach has become the dominant mode of PHC for many countries. (ii) Primary care: refers to the entry point into the health system and the place for continuing health care for most people, most of the time. (iii) Comprehensive PHC: a strategy for organizing health care systems and society to promote health. This approach is based on the Alma Ata Declaration. It has been implemented in few countries and in several small-scale experiences, although is mentioned as the goal for most of the PHC-based reforms (2).

### Case selection process

Cochabamba was selected as an exemplary of a selective PHC approach, Vicente López as an example of a primary care approach and Rosario as an example of the comprehensive PHC approach (2,3).

### Data collection processes and analysis techniques

The research included primary and secondary data source analyses and multiple data collection techniques such as documental and official record reviews, semi-structured interviews and organizational and household surveys (Table 2). We used the same data collection procedures and tools in each case, with minor adaptations as required. Some techniques were combined sequentially and others complementary.

**Table 2.** Analytical categories by dimension, definition and data source

Dimension	Category	Category’s conceptual or operational definition	Data source					
			Document review	Interviews with policymakers	Interviews with primary level and hospital managers and providers	Secondary sources review	Organizational survey	Household survey
Institutional process	Institutional structures	Regulatory structures formally or factually introduced by the reform to act upon the system of health services, such as policies, programmes and management structures	X	X				
	PHC approach envisioned	The PHC approach is characterized as selective PHC, primary care or comprehensive PHC	X	X	X			
	Changes promoted	Health care and organizational changes promoted by the institutional structures	X	X	X			
Collective action	Composition	Key actors, taking action to realize the changes promoted by the reforms		X	X			
	Relationship	The relationship between the actors comprising the collective action is characterized as competitive, collaborative or neutral		X	X			
	Leadership	Actor who leads the collective action decision-making processes		X	X			
	Outsider actors	Identification of the key actors who are outsiders to the collective action		X	X			
	Relationship with outsiders	The relationship between the collective action and key actor outsiders is characterized as conflictive, collaborative or neutral		X	X			
	Technical capacities	Ability of the collective action to create or manage regulatory mechanisms that emerge from the new institutional structures (9,13)		X	X			
	Political capacities	Ability to achieve the social and professional legitimacy required to create and carry out new institutional structures (9,13)		X	X			
Reform achievements	Physical structure and human resources	Changes in the number of primary level health centres, team composition and office hours’ coverage		X		X	X	
	Access to primary health care services	Annual average of medical visits to a primary health care centre among children under age 11						X
	Continuity of care/affiliation to the primary level of care	Percentage of children under age 11 that have a regular source of care in a primary health care centre						X
	Coordination of services between health care levels	Percentage of health centres managing patient referrals to hospitals and support services					X	
	Scope of institutional change	Influence of the changes on the entire health system characterized as *peripheral* (introduction of new practices and/or programmes limited to a primary level that is subordinate to and/or isolated from the rest of the system); *intermediate* (strengthening of primary health care with significant changes in the health care and organizational models that question the system status quo); or *strategic* (introduction of changes in the system’s health care and organizational models that locates the primary health care level at the core of the system)		X	X			
							

First, we conducted a document review of norms, policy documents and scientific literature to define reform periods by identifying new institutional structures. The document review was validated by and used to inform interviews with different actors. Second, to characterize the institutional process and the collective action during each period, we conducted semi-structured interviews with policymakers and with primary level and hospital managers and providers. Overall, we conducted 156 semi-structured interviews, including 18 with policymakers (e.g. health secretaries, PHC managers) and 138 with hospital and PHC centre managers and health care providers. Interviews were recorded, transcribed *verbatim*. A directed content analysis approach was used in documents and interviews. This approach consists in using a theory as guidance for coding. We draw our coding based on dimensions and categories of analysis relevant to the structuring process (described in the next section). To strengthen reliability, two researchers independently conducted the analysis and then contrasted and discussed the differences in their findings. Third, we conducted an organizational survey of all PHC centres in each municipality to gather data on the structure and organizational practices at the primary level. The questionnaire, administered by a researcher, included questions about the date the health centre was created and its current resources and organizational practices. We reviewed official records about the structure of the services to complete missing data when necessary. We used the survey results to categorize the health centres into three groups according to their structure and functioning and then selected two health centres per category in each municipality in consultation with policymakers. Fourth, we conducted a household survey to determine the PHC achievements in each municipality. We applied the survey to age- and sex-stratified sample of 450 caregivers of children under 11 years of age living in the area of influence of the health centres. We used descriptive statistical analysis to analyse both surveys. Finally, to validate and enrich our analysis, we organized workshops with policymakers and managers to present and discuss our findings.

### Study analytical categories by dimension

This study comprises three dimensions of analysis: the institutional process, the collective action and the reform achievements. Each dimension was approached with using a set of analytical categories or subdimensions. Table 2 shows each dimension in the first column and its analytical categories in the second column. In the third column, we provide the categories’ conceptual or operational definitions. The next columns indicate the data sources (the data collection technique/s are described in the previous subheading) used to study each category.

The first dimension, the institutional process, was studied through three categories: (i) identification of the institutional structures introduced by the reforms; (ii) PHC approach envisioned by the institutional structures; and (iii) health care and organizational changes promoted by the institutional structures. The second dimension, the collective action, was characterized in terms of seven categories: (i) collective action composition; (ii) internal relationships between their participants; (iii) actor that exerted the leadership role (11); (iv) stakeholders external to the collective action; (v) relationship between the collective action and outside stakeholders; (vi) collective action’s technical capacities; and (vii) collective action’s political capacities to implement and manage new institutional structures (10,15,16). The third dimension, the reform achievements, was characterized by five categories: (i) changes in the physical structure and human resources of the primary level of care; (ii) access to the primary level of care; (iii) continuity of care, in terms of the affiliation of the population to the primary level of care; (iv) coordination of services, in terms of the capacity of the primary level of care to manage access to specialized practices or visits with hospitals; and (v) scope of the changes in the entire health system (17). Given the lack of information about the characteristics of the structure and performance of services during the entire process, it was only possible to reconstruct the characteristics of the structure at the beginning and end of the study period and to determine the performance of the services at the end of the study period, as shown in Table 4.

### Analytic strategy

Temporal bracketing was applied as a sense-making strategy (18). The concept of temporal bracketing is derived from Anthony Giddens’ structuration theory (19), which states that actors’ actions are constrained by social structures and that actions simultaneously reconstitute those structures over time. Because mutual influences are difficult to capture simultaneously and because changes in structures follow diachronically from action, temporal bracketing proposes to analyse these interacting dimensions by temporarily ‘bracketing’ one of them. Thus, we defined periods by identifying the introduction of new institutional structures. In each period, we analysed the institutional process, the collective action and when possible, the health care and organizational changes occurred in system of health services as a result of the reform.

## Results

In this section, we describe the implementation of the processes in each case under study. We begin with an overall description of the institutional structures created, their main objectives, the characteristics of each collective action, the scope of the achievements (Table 3) and the changes in the primary level structure and health care performance at the end of the study period (Tables 1 and 4). Then, we provide a detailed description of the health care and organizational practices promoted by the new institutional structures introduced during each period and the changes in the composition of the collective action, including internal and external relationships and political and technical capacities (Table 3).

**Table 3.** Description of the institutional process, the collective action and the scope of the institutional change, by case and periods

Cochabamba					
Dimensions	Categories	Periods: 1993–96	1996–2003	2003–08	
Institutional process	Institutional structures	Popular Participation Law Grassroots Territorial Organizations Local Health Directorates	Universal Mother and Child Insurance Creation of Municipal Health Directorates in charge of managing municipal primary health services	Family, Community and Intercultural Health Strategy	
	PHC approach envisioned	Shift from a curative and hospital-centric model to a selective PHC approach	Selective PHC	Comprehensive PHC	
	Changes promoted	Basic package of services with emphasis on prevention targeting maternal and child populations Popular participation Economic incentives and resource transfers from national level to municipalities	Expansion of the maternal and child service package Popular participation Introduction of health services management and norms	Integration of indigenous and popular medicine Expansion of the service package beyond maternal and child services Popular participation	
Collective action	Composition	Grassroots Territorial Organizations and municipal and departmental health managers	Grassroots Territorial Organizations and municipal and departmental health managers	Grassroots Territorial Organizations and municipal and departmental health managers	
	Relationship	Competitive	Competitive	Competitive	
	Outsider actors	Primary level and hospital practitioners	Primary level and hospital practitioners	Primary level and hospital practitioners	
	Relationship with outsiders	Conflict-filled. Disputes around who should manage the PHC services and the regulation of physicians’ working hours	Conflict-filled. Disputes around the definition of professional physicians practices	Conflicts with western- minded professionals and physicians against the Family, Community and Intercultural Health Strategy conceptions	
	Leadership	Grassroots Territorial Organizations	Grassroots Territorial Organizations	Grassroots Territorial Organizations	
	Technical capacities	Technical difficulties implementing the measures promoted by the national reform	Difficulties managing the purchase and management of medicines and medical supplies	Weak capacities for introducing the Family, Community and Intercultural Health Strategy into health services	
	Political capacities	Strong social legitimation supported by population and social movements advocating for improved health access Lack of professional legitimation; physicians opposed to the reform	Strong social legitimation supported by population and social movements advocating for improved health access Lack of professional legitimation; physicians opposed to the reform	Strong social legitimation supported by population and social movements advocating for improved health access and respect for and recognition of indigenous culture Lack of professional legitimation; physicians opposed to the reform	
Reform achievements	Scope of the institutional change	Peripheral	Peripheral	Peripheral	
Vicente López					
Dimensions	Categories	Periods: 1993–96	1996–2003	2003–08	
Institutional process	Institutional structures	PHC municipal department and programmes targeted at the maternal– child population with support from the provincial Ministry of Health and the Pan American Health Organization	Strengthening of the institutional structures emerged in the previous period	Spaces of informal coordination across health care levels	
	PHC approach envisioned	Shift from a curative and hospital-centric model to a selective PHC approach	Primary care	Comprehensive PHC	
	Changes promoted	Community participation Improvement health care access Emphasis on health care promotion and preventive practices	Expansion of practices and services covered and strengthening of affiliation to the primary level Strengthening of the primary level of care’s problem-solving capacity and technical quality	Coordination between health care services and professional agreements between primary care and hospital physicians Strengthening of the primary level of care’s problem-solving capacity and technical quality Increase in the proportion of multidisciplinary PHC teams Strengthening of the figures of health centres and programme coordinators	
Collective action	Composition	PHC Department, primary level practitioners and social movements	PHC Department, primary level practitioners and social movements	PHC Department and primary level practitioners	
	Relationship	Collaborative	Competitive	Collaborative	
	Outsider actors	Hospital physicians and managers	Hospital physicians and managers	Hospital physicians and managers	
	Relationship with outsiders	Neutral	Competitive with hospital physicians	Collaborative with hospital physicians and competitive with social movements	
	Leadership	PHC Department	PHC Department	PHC Department	
	Technical capacities	Teams with managerial and professional expertise and Pan American Health Organization and Provincial Ministry support	Teams with managerial and professional expertise and Pan American Health Organization and Provincial Ministry support	Teams with managerial and professional expertise	
	Political capacities	Social and professional legitimation supported by population and social movements advocating for improved access to health services	Professional legitimation thanks to the primary level practitioners’ support	Professional legitimation thanks to the primary level practitioners’ support and the strengthening of the management structure	
		Primary level practitioners who share the reform principles and have been strengthening their professional role with the proposed changes	Decreased support from population and social movements	Decreased social legitimation and population support becomes irrelevant	
Reform achievements	Scope of the institutional change	Peripheral	Peripheral– intermediate	Intermediate	
Rosario					
Dimensions	Categories	Periods: 1990–94	1995–2000	2001–04	2005–08
Institutional process	Institutional structures	PHC Department. Maternal and child programmes	PHC districts	Health districts	Matrix- management model
	PHC approach envisioned	Shift from a curative and hospital-centric model to a selective PHC approach	Primary care	Comprehensive PHC	Comprehensive PHC
	Changes promoted	Health care and organizational regulations at the primary level	Transfer of health care practices to the primary level Participatory management of the primary level Geographic integration of primary level services under the PHC districts Geographic assignment of the population to health centres	Integration of levels of care. Establishment of the primary level as a gateway and as the coordinator of the care process Regulation of access to specialized practices and services	Articulation of professionals from different levels for the development of case management agreements and care guidelines
Collective action	Composition	PHC Department, primary level practitioners and social movements	PHC Department, primary level practitioners and social movements	PHC Department and health care system managers, primary level practitioners and social movements	PHC Department, policymakers and health care system managers, primary level practitioners and social movements
	Relationship	Collaborative	Collaborative	Collaborative	Collaborative
	Outsider actors	Hospital specialist physicians	Hospital specialist physicians	Hospital specialist physicians	Hospital specialist physicians
	Relationship with outsiders	Neutral	Competitive	Collaborative	Collaborative
	Leadership	Primary level physicians	Primary level teams and managers	Service managers	Policymakers
	Technical capacities	Primary care managers with expertise in health care management	Primary care physicians trained in postgraduate general medicine programmes	Increased managerial and health care expertise	Increased managerial and health care expertise
	Political capacities	Social legitimation due to social movement support Professional legitimation due to primary level physicians support	Social legitimation due to social movement support Professional legitimation due to primary level teams support and academic and professional development	Professional legitimation due to the integration of the primary level teams and managers into health service managers’ positions beyond the primary level	Professional legitimation due to the firm rooting of the primary level teams and managers in health system decision maker positions
				Social legitimation due to social movement support and recognition of the movement’s achievements by hospital physicians	Social legitimation due to social movement support and recognition of the movement’s achievements by hospital physicians
Reform achievements	Scope of the institutional change	Peripheral	Intermediate	Strategic	Strategic

Source: Own data collected based on primary sources (interviews and workshops) and secondary sources (document review) as part of this research.

**Table 4.** Reform achievements in terms of changes in the structure and health care performance of the primary level of health care

	Changes in the structure of the primary level*						Service performance of the primary level (2008)^a^				
	Number of primary care health centres		% of primary health care centres with a multidisciplinary team^b^		Primary health care centres’ office hours per week (average)		Access to primary health care services**		Affiliation with the primary level of care**		Coordination of services between health care levels*
	At start of each reform****	2008	At start of each reform***	2008	At start of each reform***	2008	Annual average of medical visits among children under age 11	Annual average of medical visits to a primary health care centre among children under age 11	% of children under age 11 that have a regular source of care^c^	% of children under age 11 that have a regular source of care in a primary health care centre	% of health centres managing patient referrals to hospitals and support services
Cochabamba	6	28	0%	0%	Reduced and unstable	35.3	3.8	1.7	20.2%	4.0%	0.0%
Vicente López	7	20	0%	60.0%	Reduced and unstable	46.9	7.9	3.9	65.1%	46.2%	30.0%
Rosario	49	78	0%	73.8%	Reduced and unstable	59.3	7.6	4.4	78.4%	55.1%	100%

^a^There are no available data prior to the change process for these variables.

^b^The composition of a team was considered to be multidisciplinary whenever the following elements were present, at a minimum: In Argentina: (i) a generalist or paediatrician, (ii) a clinician, a gynaecologist or ob-gyn, (iii) a nurse and (iv) a social worker or a psychologist. In Bolivia: (i) a GP, (ii) a dentist, (iii) a licensed nurse and (iv) an auxiliary nurse.

^c^A regular source of care is considered to be available whenever there is a source of care or physician that regularly provides medical care to.

Source: Authors’ interpretation based on: *Organizational survey: Rosario, 2007; Vicente López and Cochabamba, 2008.

**Household survey: Rosario, 2008; Vicente López and Cochabamba, 2008/2009.

***Interviews with PHC policymakers and managers.

****Start of each reform: Cochabamba 1993; Vicente López 1993; Rosario 1990.

### Cochabamba

In Cochabamba, we identified three stages in the national reforms in order to transfer resources to the municipalities; create new local structures and economic incentives to promote popular participation; decentralize the management of health services to the municipalities; and expand coverage and access to health services. The first two stages envisioned an improvement in coverage and access to services for the maternal–child population and the final stage focused on the development of a comprehensive PHC approach based on a multicultural model. In this case, collective action was composed of social movements (which had a leadership role) and municipal and departmental (provincial) health managers, who were engaged in a competitive relationship with each other and with health providers from the primary level of care. The reform achieved the development of a structure for the primary level of care with the creation of 22 new health centres that offer health services, mainly preventive and maternal–child care, provided during limited operating hours and without the capacity to coordinate their patients’ care processes with hospitals (Tables 1 and 4). This development enabled the channelling of 44.7% of the total visits with children under age 11 to the primary level of care. However, the annual rate of visits in this age group was lower than the two recommended annual visits (mainly well-child check-ups) and only a reduced percentage of children had a regular source of care at the primary level of care (Table 4), due to the low ratio between health centres and physicians per inhabitants without coverage (Table 1). This stage is described below.

During the first stage, the reform creates the Grassroots Territorial Organizations (Organizaciones territoriales de base—OTB) and the Local Health Directorates (Directorios Locales de Salud—DILOS). The Local Health Directorates were tripartite structures comprised the Grassroots Territorial Organizations (head of the Local Health Directorates), the municipal and the departmental health authorities. The Local Health Directorate of Cochabamba is left in charge of the primary health care centres. Previously, the primary health care level was dependent on the hospitals and the departmental authorities, and in each health centre, the managerial functions relied on the main physician.

The Local Health Directorate embodies the collective action. Its actions served as the foundation for both expanding the structure of the primary level of care with the creation of seven new health centres and improving the coverage of preventive services for the maternal–child population. However, these actions took place in a framework of internal conflict-filled relationships between physicians, who perceived that their power over services and their professional autonomy was threatened. The strength of collective action resided in the political capacities derived from the social legitimacy that emerged from the support of social movements (which provided physical spaces for expanding the primary level of care). Its weaknesses were the lack of professional legitimacy and technical capacity to manage health services.

The second stage was defined by the creation of the Universal Mother and Child Insurance (Seguro Universal Materno Infantil—SUMI), which sought to expand free health services for the maternal–child population and integrate new norms of health services management and care provision. At the local level, the Municipal Health Directorate (Directorio Municipal de Salud—DIMUSA) was created and put under the charge of the local management of health services. Here, nine health centres were inaugurated. The composition, relationships and capacities of the collective action continued unchanged with respect to the previous stage.

The third stage was structured around the creation of the national-level Family, Community and Intercultural Health Strategy (Salud Familiar Comunitaria Intercultural—SAFCI), which sought to expand the coverage of health insurances beyond the maternal and child population and promote a comprehensive PHC approach with a focus on multiculturality. During this stage, six new health centres were added to the primary level of care. Although the conflicts within the collective action decreased here, the relevant changes in the composition, relationships and capacities of collective action did not take place.

### Vicente López

The reform process in Vicente López was developed in three stages that created formal and informal institutional structures aimed at consolidating a primary level of care with the capacity to provide comprehensive, high-quality health care. Collective action here was led by the managers of the PHC Department in collaboration with primary level of care professionals and social movements. These social movements gradually subsided during the second and third stages. Given that hospital managers and professionals did not share the reform values, they remained distant and indifferent during the first stage. Nevertheless, during the second stage and with the attempt to expand the practices envisioned for the primary level of care, a dispute emerged between health professionals from different levels of care. At the beginning, the reform envisioned a selective PHC approach, which provided the foundation for the gradual creation of an autonomous, relatively strong and comprehensive primary level of care. During the reform, the number of primary level health professionals increased and 13 new health centres were created. This led to a relatively high ratio of health centres and physicians per inhabitants without coverage at the end of the study period (Table 1). By 2008, more than half of the centres provided a wide range of health services during extended operating hours, but with limited capacities to coordinate their patients’ care processes with hospitals (Table 4). With this development, 46.2% of the total number of children under age 11 had a regular source of care in the primary level of care. This level of care represented 49.4% of total visits and achieved a rate of 3.9 annual visits in this population (Table 4). Each stage of this change process is described below.

During the first stage, the Municipal PHC Department was created with support from the provincial Ministry of Health and the Pan American Health Organization/World Health Organization (PAHO/WHO). The Municipal PHC Department opened four new health centres and developed different health promotion and prevention programmes, targeted mainly towards the maternal–child population and infectious diseases. The political capacities of this Department were embedded in professional legitimations provided by the primary level professionals who shared the reform principles and benefitted from the reform and by the provincial Ministry of Health and PAHO/WHO who supporting and strengthening the technical capacities provided. Additionally, the social movements that advocated for improved health care access were well-channelled and therefore provided both social legitimation for the reform and physical spaces for expanding the structure of the primary level of care.

During the second stage, the PHC Department strengthened the structure of the primary level of care with six new health centres and expanded the health programme beyond maternal–child health and infectious disease. The Department also promoted expanded health practices, improved technical quality and strengthened role for the primary level of care as both a regular provider of care and the coordinator of the population’s care processes. Within the collective action, there were disputes with the social movements who wanted to influence the health services functioning in their facilities. There were also disputes with hospital specialists as the teams of the primary level services expanded their composition, practices and competencies.

The third stage created informal spaces for coordination between hospital heads of service, health centre directors and PHC programme coordinators. During this stage, the conflicts with hospitals declined and three additional health centres were created. The social movements were excluded from the collective action, despite the contributions that they made by providing infrastructure support for the creation of new health centres.

### Rosario

In Rosario, there were four periods marked by new managerial and decision-making structures created by the Municipal Secretary of Public Health. These structures favoured the strengthening and empowerment of health services management through a PHC movement made up of workers from the primary level of care. Using these structures, the PHC movement was able to achieve enhancements in the structure and organizational and health care practices of the primary level from the outset. The movement then gradually consolidated the primary level as a gateway and as the coordinator of the care process and enabled the principles and practices of the comprehensive PHC approach to permeate the other levels of the system. The collective action was led by a municipal movement of mostly made up of primary level professionals (practitioners and managers) who were embedded in service management structures, in partnership with social movements. The reform achievements radically improved the structure and organization of the primary level of care and introduced a PHC-based transformation into the entire health system. During the reform, 29 new health centres were created, adding to the 49 centres that existed prior to the reform. This led to the result of a relatively high ratio of health centres and physicians per inhabitants without coverage (Tables 1 and 4). Approximately 75% of these centres provided a wide range of services that were integrated with hospitals and available during extended operating hours (Table 4). With this development, the system achieved that 55.1% of all children under age 11 had a regular source of care at the primary level. This channelled 57.9% of the total number of visits and led to a rate of 4.4 visits per year in this population. Each stage of the change process is described below.

During the first stage, the PHC Department was created. The Department developed preventive programmes targeted at the maternal–child population and introduced management norms to regulate operating hours, medical records and the use of supplies in health centres. At this stage, 25 new health centres were created. The participation of professionals with experience in service management provided technical capacity for the collective action. The political capacities emanated from the social legitimacy provided through support from social movements that sought to improve the population’s access to health care and from the professional legitimacy based on a participatory management model of professionals with strong personal leadership skills. During this stage, hospital managers and professionals were indifferent to what was happening in the primary level.

The second stage created PHC districts and participatory management structures and decentralized the coordination of health centres. Health teams and social movements were invited to participate in these initiatives. Health centres were assigned responsibility for a geographically defined population and their practices were expanded (e.g. health centres were given responsibility for prenatal check-ups, which were previously provided at hospitals). Nine new health centres were created, new professional profiles were integrated into health centres and the range of services offered at the primary level was expanded. This advance in service delivery that was formerly the exclusive competency of specialists led to a dispute with hospitals. The PHC movement created a postgraduate programme in general medicine, which strengthened the technical capacity and professional legitimacy of the collective action.

The third stage created health districts that generated spaces for articulation between the PHC districts and hospitals. During the economic crisis that dominated this stage, several health centres operating within community organizations closed. The need to manage limited resources more efficiently favoured the regulation of access to the second level of care, in turn strengthening the figure of the primary level as a gateway and as the coordinator of the population’s care processes and blurring the dispute with hospitals. This role strengthened the social and professional legitimacy of the collective action, supported by a process of accumulation of professional, academic and political prestige.

During the fourth stage, the PHC movement—which was embedded in the management structures of the second level of care—implemented a matrix-management model. This model established processes for collaboration between professionals in the primary and second levels of care in a determined geographic area with the goal of strengthening the comprehensiveness and technical quality of the primary level (through the joint development and implementation of care guidelines), the continuity of the care process, coordination between providers and access to specialized practices. The collective action established collaborative relationships with hospital professionals and their technical and political capacities continued improving. The number of health centres recovered with the construction of municipal health centres.

## Discussion

In this section, we interpret our findings and attempt to identify the enablers of the collective action capacities to produce (or not produce) the results envisioned in each case under study. In doing so, we consider the collective action capacities to be the result of the dynamic of the structuring processes between the institutional structures and the collection action.

In Cochabamba, the new structures produced at the national level resulted in weak collective action, with internal conflicts and confrontation with the physicians in the system. This dynamic impeded the strengthening of the technical and political capacities needed to exploit new institutional structures, guarantee effective implementation of the reform and provide continuity for the change process. Although the reform was able to strengthen the primary level of care and introduce organizational changes and improvements in service performance, it produced a dynamic of ‘interrupted’ change limited to the development of a selective PHC approach.

In Vicente López, the new institutional structures favoured the generation of a new collective action based on the participation of professionals, managers and technical personnel from the primary level of care. This collective action was able to produce new institutional structures that expanded their technical capacities, but under a dynamic of path-dependency (20). This dynamic reproduced the development of organizational capacities and changes centred on the primary level of care but with limited influence on the rest of the health service system.

In Rosario, the new institutional structures led to the generation of a broad-based, collaborative, collective action that in turn produced these new institutional structures. These structures favoured the consolidation of greater technical and political capacities that allowed the collective action to conquer key management spaces within the health service system (21). This dialectic relationship between actors and institutional structures favoured the system’s ability to overcome technical and political challenges at each stage, enabling the consolidation of a comprehensive PHC approach.

Lessons learned from the evidence indicate that reform processes aimed at producing health systems based on a PHC comprehensive approach require collective action with a dynamic composition that includes actors from different institutional fields with capacities to confront technical and political challenges during the different stages of the change process (22). The new institutional structures should be conceived as technical mechanisms of regulation for implementing new approaches of PHC among the actors involved in the health service system. These structures also represent a platform that facilitates the emergence and/or consolidation of partnerships that foster a growing influence on the health service system.

Although this study produced significant information and findings based on a broad set of data collection techniques and a comprehensive analysis approach, it has important limitations. First, an analysis of the effectiveness of the studied reforms was outside of the scope of this study. Second, we did not analyse how the socio-economic differences between settings influenced the actors’ capacities. Third, our observation and analysis centred on the actors involved and the processes that took place at the local level and within the boundaries of the public subsystem. Little or no attention was given to the potential influence of political and institutional processes at higher government levels or in other health subsystems. Future research could shed light on these issues.

## Conclusion

The reform processes introduced new institutional structures that permitted the agglutination of different stakeholders who were potential beneficiaries of the reform as a common force, although not necessarily with the same objectives. These processes achieved an evolution from selective to more comprehensive PHC approaches, strengthening managerial capacities and expanding and improving the performance of the primary level of care. However, these changes were of differing scope depending on the capacity of the different collective actions, which represent both the product and catalyst of change in the new institutional structures.

In each case, the interaction between the characteristics of the institutional structures and the collective action produced different structuring dynamics that served as either facilitating or limiting factors in the development of the technical and political capacities that are needed to develop a comprehensive PHC approach. In Cochabamba, there was an ‘interrupted’ structuring process that achieved the establishment of a primary level with a selective PHC approach. In Vicente López, there was a ‘path-dependency’ structuring process that permitted the consolidation of a ‘primary care’ approach, but with limited influence in hospitals. In Rosario, there was a ‘dialectic’ structuring process that favoured the development of the capacities that are needed for the consolidation of a comprehensive PHC approach.

The path towards health service systems centred on PHC is neither a linear nor a natural process. The analysis of ‘structuring’ processes allows for the tracking (and potentially interventions in) of the generation of capacities for collective action (19).

## Declaration

Funding: International Development Research Centre (IDRC). The analysis that led to this paper was conducted thanks to the support of the CONICET, and the postdoctorate fellowships granted to Ernesto Báscolo by the National School of Public Administration (École Nationale d’Administration Publique) and the Department of Family Medicine at the McGill University.

Ethical approval: International Development Research Centre (IDRC).

Conflict of interest: none.
